# A review of the prevalence, associated factors and interventions of psychological symptoms among cancer patients in the Chinese Mainland during the COVID-19 pandemic

**DOI:** 10.3332/ecancer.2021.1332

**Published:** 2021-12-09

**Authors:** Binbin Xu, Marques S N Ng, Winnie K W So

**Affiliations:** The Nethersole School of Nursing, The Chinese University of Hong Kong, 6/F, Esther Lee Building, The Chinese University of Hong Kong, Hong Kong, China

**Keywords:** COVID-19, carcinoma, psychology, China

## Abstract

The novel coronavirus disease 2019 (COVID-19) has placed tremendous pressure on public health systems across the world. Compared with the healthy population, cancer patients are more prone to developing psychological problems, including depression and anxiety, because of worries about cancer recurrence, cancer symptoms, treatment-related discomfort, a lack of social interaction and the impact on their financial well-being. This paper aimed to identify existing evidence on psychological symptoms and their associated factors among cancer patients in the Chinese Mainland during the COVID-19 pandemic, and on interventions to effectively manage these symptoms. Articles related to the prevalence, the risk factors and interventions of psychological symptoms among cancer patients in the Chinese Mainland during COVID-19 published between December 2019 and August 2020 were searched in two English (PubMed and Embase) and two Chinese (CNKI and Wan Fang Data) databases. A total of 180 studies were identified, and 18 studies were included in the review after removing duplicates and screening for relevancy. The results suggest that patients with cancer in the Chinese Mainland have suffered psychological pressure during COVID-19, with a high prevalence of psychological distress, depression and anxiety reported across most of the reviewed studies. Pandemic-related factors such as treatment discontinuation and worry about being infected are associated with these symptoms. Nurses may help to relieve these symptoms by identifying stressors, providing relevant information through mass and social media and referring patients to specialists for psychological support. However, evidence about treatments and interventions for these symptoms is limited, and additional research is warranted to identify effective interventions to promote resilience in this patient population.

## Background

The novel coronavirus disease 2019 (COVID-19), which was declared a pandemic in March 2020, has placed tremendous pressure on public health systems across the world [1]. Reports suggest that the general population is experiencing increased distress related to the pandemic [2]. Cancer patients are more prone to developing psychological problems than the healthy population [3–5] and are at increased risk of depression and anxiety because of worries about cancer recurrence, cancer symptoms, treatment-related discomfort, a lack of social interaction and the impact on their financial well-being. As China has been greatly affected by COVID-19 since its outbreak in December 2019, Chinese cancer patients are experiencing increased psychological symptoms, and there is a need to improve psychological care for Chinese cancer patients at this time [6]. Therefore, this paper aims to identify existing evidence on psychological symptoms and their associated factors among cancer patients in the Chinese Mainland during the COVID-19 pandemic, and on interventions to effectively manage these symptoms.

## Methods

A literature search was conducted to identify articles related to the prevalence, associated factors and interventions of psychological symptoms among cancer patients in the Chinese Mainland during COVID-19 published between December 2019 and August 2020. Given the exploratory nature of this review, all types of articles (e.g. empirical studies, discussion papers) were considered. Two English (PubMed and Embase) and two Chinese (CNKI and Wan Fang Data) electronic databases were searched. The search terms used included: (Cancer OR oncology OR malignant tumour) AND (Psychological OR mental) AND (severe acute respiratory syndrome coronavirus 2 OR SARS-CoV-2 OR coronavirus disease 2019 OR COVID-19 OR 2019 novel coronavirus OR 2019-nCoV). A total of 180 studies were identified. After removing duplicates and screening for relevancy to the review topic, three studies in English [7–9] and 15 studies in Chinese [10–24] studies were included in the review.

## Results

### Prevalence of psychological symptoms

Three main psychological symptoms, including psychological distress [7, 8, 11], anxiety, and/or depression [7–10, 13–15, 17, 22] were identified among patients with cancer in the Chinese Mainland during COVID-19.

In one study of 189 patients with cancer in Guangdong province, psychological distress was reported by 50.8% of participants [11]. Patients who perceived a very serious impact of the COVID-19 pandemic on investigation/treatment (*F* = 7.734, *p <* 0.001), daily activities (*F* = 12.982, *p* < 0.0001) and family economy (*F* = 7.001, *p <* 0.001) had a higher level of distress than those who perceived no or less serious impact [11]. In another study of 628 patients with breast cancer in Hubei Province (pandemic epicentre) [8], more than half (52.3%) of the sample reported a moderate to severe level of distress. The study revealed that having been to Wuhan (the original epicentre of the pandemic), the discontinuation of cancer treatment, perceived poor health and a diagnosis of cancer metastasis were possible reasons for the elevated distress. A third study assessed psychological distress in terms of fear of disease progression [7]. This fear was identified in 86.5% of 326 patients with cancer in Wuhan, especially those with lung cancer and those who had experienced delays in or the discontinuation of treatment.

Depression and anxiety are common psychological symptoms associated with cancer. Several of the studies [7–10, 13–15, 17, 22] assessed these symptoms in various populations of patients with cancer in Chinese Mainland.

### Impact of geography

Among breast cancer patients, one nationwide study found that 40.9% had depression and/or anxiety [10], and another study in Hubei province (which includes the city of Wuhan) reported a higher prevalence of depression and anxiety of 47.3% and 56.2%, respectively [8]. In studies of other populations with cancer, a wider variation was observed. High prevalence of depression (50.4%–74.5%) and anxiety (53.5%–67.5%) were found in two mixed samples in Wuhan [7, 9]. Slightly lower rates of anxiety (45.5%–53.3%) were reported among patients with cancer in Sichuan and Xi’an [14, 15].

### Impact of disease

In terms of specific cancer types, one study found that 63.4% and 70.0%, respectively, of patients with haematological malignancies, reported depression and anxiety [22]. Among them, 14.7% and 12.0%, respectively, reported severe symptoms. In another nationwide study of lung cancer patients [17], the prevalence of depression and anxiety were 52.6% and 74.6%, respectively.

Only one study examined psychological symptoms among patients with cancer who underwent specific treatments during the COVID-19 pandemic [13]. The findings showed that 27.7% and 15.8% of 101 patients who received radiotherapy reported depression and anxiety, respectively, and the proportion that had severe symptoms was low (3.0% for depression and 1.0% for anxiety) [13].

Some of the above-mentioned studies explored factors related to depression and anxiety. In one study [8], these symptoms were associated with a history of being in Wuhan. In addition, patients who had had their cancer treatment discontinued or central venous catheter (CVC) flushing delayed had an increased risk of both symptoms. Other studies found similar links between anxiety and depression and discontinued treatment [7, 10, 15]. One study found that being an outpatient and a lack of knowledge about COVID-19 prevention were associated with anxiety [22]. In other studies [7, 15], patients who shared concerns about the pandemic reported higher levels of depression and anxiety. Other factors associated with depression and/or anxiety included being female [22], being unmarried [7, 22], having a lower income [7, 22], having perceived poor health [22], having lung cancer [7], a short duration since diagnosis (≤1 year) [8], not staying in hospital [22] and having a CVC *in situ* [22].

### Psychological interventions for patients with cancer

Evidence of effective psychological interventions for cancer patients during COVID-19 remains limited. The interventions described in most of the identified studies were based on the authors’ clinical experience and expertise.

Some studies reinforced the significance of routine assessment and psychological self-care. One study [16] recommended the use of a ‘daily symptom monitor’ and other questionnaires to evaluate and report psychological symptoms. ‘Self-care’ refers to encouraging patients to have a healthy lifestyle, maintain social contact, perform leisure activities, apply relaxation strategies, and seek help for psychological support [16, 21].

In terms of cancer care, one study [20] suggested that symptom management and psychological support need to be strengthened to alleviate patients’ suffering. Because clinical services have been severely disturbed during the pandemic, telephone follow-ups, online consultations and community care services could help to fill the gap [19, 23]. In addition, healthcare professionals could provide relevant information through the mass media (e.g. newspapers, websites) and social media to ease concerns associated with inadequate knowledge about COVID-19 [23, 24]. One study [19] proposed a three-tier system to enhance support for cancer patients: 1) government level, which would provide accurate information and appropriate services to support cancer patients; 2) hospital level, which would offer online services and individual psychological care; and 3) personal level, which would enhance awareness of psychological well-being and self-care.

Two studies on specific interventions were identified. One study found a significantly lower anxiety score (from 54.5 ± 9.3 to 49.6 ± 8.8; *p* = 0.002) among oncology in-patients (*N* = 50) who received health education about COVID-19 that was delivered by nurses [18]. The other evaluated the effectiveness of a ‘nurse-led intervention’ for stress reduction [12], in which nurses used telephone calls and home visits to identify stressors in patients’ narratives and invited two experts in psychological counselling to guide the intervention. This approach significantly reduced the ‘distress thermometer score’ from 3.69 ± 1.97 to 2.02 ± 1.75 out of 10 (*p* < 0.05). In addition, the proportion of patients with depression (from 74.2% to 50.0%) and anxiety (from 22.6% to 9.7%) significantly decreased (all *p* < 0.05). However, the findings of this study were limited by small sample size (*N* = 62) and a lack of controls.

## Discussion

This review suggests that patients with cancer in Chinese Mainland have suffered psychological pressure during COVID-19, with a high prevalence of psychological distress, depression and anxiety reported across most of the reviewed studies. Pandemic-related factors such as treatment discontinuation and worry about being infected are associated with these symptoms. However, evidence on effective interventions for these symptoms is lacking.

The prevalence of psychological symptoms in the identified studies was higher than those found in the general public (29.6% for stress, 33.7% for depression and 31.9% for anxiety) [2]. In addition to cancer itself, this difference may be associated with the challenges encountered by cancer patients during COVID-19. Because clinical services have been greatly affected by infection control measures and manpower shortages [25], cancer treatments have been delayed or discontinued. Several of the studies suggested that this situation has led to adverse psychological outcomes [7, 8, 10, 15], and proposed measures to overcome the problem, including telephone follow-ups and online consultations. These measures were not found to be inferior to face-to-face consultations and received positive feedback [26].

This review shows that the experience of depression and anxiety varies across cancer types [17, 22]. For patients with the same type of cancer, the prevalence of depression and anxiety reported by different studies [8, 10] also varies. It may be explained by the instrument used (e.g. the Generalised Anxiety Disorder Questionnaire or the Hospital Anxiety and Depression Scale) to assess psychological symptoms. Besides, one study [13] found that the intensity level of psychological symptoms among the participants receiving radiation treatment was relatively low, which may be explained by the heterogeneity of the sample. Of note, the study site and time period may have affected the results. Patients in studies conducted in Wuhan [7, 9] or during the early stage of the pandemic [22] might have experienced greater uncertainty related to the pandemic, which may have been associated with depression and anxiety.

Our findings also suggest that patients in the community with inadequate knowledge of or elevated concerns about COVID-19 have a higher risk of depression and anxiety, as demonstrated by a large-scale study in Wuhan [27]. Clearly, information provision is important in minimising the psychological impact of COVID-19. While it is the responsibility of the government to provide up-to-date and accurate information about the pandemic, healthcare professionals could disseminate this information through popular channels such as WeChat to increase receptibility.

Finally, our findings show that high-quality evidence about psychological interventions for cancer patients during the pandemic is lacking. Although China is rapidly recovering from the COVID-19 pandemic, there is still an urgent need to provide psychological support to promote resilience and prevent long-term complications, such as post-traumatic stress disorder, in cancer patients [28]. Additional research is warranted to examine the feasibility and effectiveness of stress reduction programs (e.g. cognitive behaviour therapy, mindfulness) in cancer populations in Chinese Mainland [29, 30].

## Conclusion

Living with cancer is a stressful experience. The outbreak of COVID-19 has brought more uncertainties and challenges to patients with cancer in the Chinese Mainland. These uncertainties threaten their psychological well-being and can lead to various psychological symptoms, including distress, depression and anxiety. A multidisciplinary collaboration involving nurses may help to relieve these symptoms.

Nurses play a vital role in assessing symptoms, providing relevant information support through mass and social media and referring patients to psychologists for specialised psychological support. However, evidence about treatments and interventions for these symptoms is limited, and additional research is warranted to identify effective interventions to promote resilience in this patient population.

## Conflicts of interest

There are no conflicts of interest.

## Funding

This research received no specific grant from any funding agency.

## Authors’ contributions

Winnie K W So conceived the aim and design of the review. Binbin Xu and Marques S N Ng performed the literature search and data extraction and drafted the manuscript. Winnie K W So critically revised the article. All authors have read and approved the final version of the article.

## References

[1] World Health Organization Timeline: WHO’s COVID-19 response. https://www.who.int/emergencies/diseases/novel-coronavirus-2019/interactive-timeline.

[2] Salari N, Hosseinian-Far A, Jalali R (2020). Prevalence of stress, anxiety, depression among the general population during the COVID-19 pandemic: a systematic review and meta-analysis. Global Health.

[3] Mitchell AJ, Chan M, Bhatti H (2011). Prevalence of depression, anxiety, and adjustment disorder in oncological, haematological, and palliative-care settings: a meta-analysis of 94 interview-based studies. Lancet Oncol.

[4] Singer S, Das-Munshi J, Brähler E (2010). Prevalence of mental health conditions in cancer patients in acute care—a meta-analysis. Ann Oncol.

[5] Song H, Li J, Lu Y (2013). Investigation of mental health and its influence on Chinese cancer patients using a multidisciplinary screening flow: an epidemiological survey in the west of China. Chin Med J (Engl).

[6] Wang Y, Duan Z, Ma Z (2020). Epidemiology of mental health problems among patients with cancer during COVID-19 pandemic. Transl Psychiatry.

[7] Chen G, Wu Q, Jiang H (2020). Fear of disease progression and psychological stress in cancer patients under the outbreak of COVID-19. Psychooncology.

[8] Li J, Cesar Augusto S, Feng H (2020). Patient-reported outcomes of patients with breast cancer during the COVID-19 outbreak in the epicenter of China: a cross-sectional survey study. Clin Breast Cancer.

[9] Qian Y, Wu K, Xu H (2020). A survey on physical and mental distress among cancer patients during the COVID-19 epidemic in Wuhan, China. J Palliat Med.

[10] Chen D, Zhang Y, Lv L (2020). A survey of treatment and psychological status of breast cancer patients during the pandemic of COVID-19. J Mod Oncol.

[11] Chen X, Chen C, Chen X (2020). Analysis of psychological distress and related factors of tumor patients during the epidemic of novel coronavirus pneumonia. China J Health Psychol.

[12] Wang F, Liu Y (2020). The application of narrative nursing in tumor patients during COVID-19 epidemic. Pract J Med Pharm.

[13] Ma J, Zhen H, Guan H (2020). Analysis of anxiety and depression in patients undergoing radiotherapy during COVID-19 epidemic period. Chin J Radiat Oncol.

[14] Qin J, Kuang Y, Liu M (2020). Discussion on the guidance of home care for cancer patients during the epidemic period of 2019 novel coronavirus pneumonia. Smart Healthcare.

[15] Shi F, Liu Z, Zhang Y (2020). Study of the anxiety status and influencing factors of tumor patients during the epidemic of COVID -19. Mod Oncol.

[16] Wu F, Lu J, Liu L (2020). Psychological intervention for patients with gynecological tumors at home during the prevention and control of the novel coronavirus (SARS-CoV-2). Prog Obstet Gynecol.

[17] Xu H, Yang K, Yang G (2020). Explore the optimal resolvent of medical needs and mental health for patients with lung cancer during epidemic novel coronavirus pneumonia. Chin J Lung Cancer.

[18] Yang Y, Zhong L, Peng L (2020). Investigation and analysis of psychological status of tumor patients during COVID-19. Chin Gen Pract Nurs.

[19] Yao H, Yang S, Zhang J (2020). Psychological state and adjustment strategy of tumor patients under novel coronavirus pneumonia epidemic situation. Mod Oncol.

[20] Zhang M, Qin H, Tao H (2020). Expert consensus on nursing care for COVID-19 prevention and control of lower gastrointestinal tumors in Guangdong Province. Guangdong Med J.

[21] Zhang Y, Luo X, Zhang J (2020). Psychological stress and protective strategies in cancer patients during the outbreak of corona virus disease 2019. J Cancer Control Treat.

[22] Zhao M, Peng D, Liu Q (2020). Investigation on mental status of patients with hematological malignancies during the COVID-19 outbreak and related influencing factors. Nurs Interg Trad Chin West Med.

[23] Yang L, Fang C, Xiong Y (2020). Clinical management strategies of patients with lung cancer during novel coronavirus pneumonia. Med Inform.

[24] Gao F, Bi X, Zhang X (2020). Health education management of cancer patients during novel coronavirus pneumonia epidemic. Mod Oncol.

[25] Wang Z, Wang J, He J (2020). Active and effective measures for the care of patients with cancer during the COVID-19 spread in China. JAMA Oncol.

[26] Greenhalgh T, Wherton J, Shaw S (2020). Video consultations for COVID-19. BMJ.

[27] Wang C, Pan R, Wan X (2020). Immediate psychological responses and associated factors during the initial stage of the 2019 coronavirus disease (COVID-19) epidemic among the general population in China. Int J Environ Res Public Health.

[28] Horesh D, Brown AD (2020). Traumatic stress in the age of COVID-19: A call to close critical gaps and adapt to new realities. Psychol Trauma.

[29] Nenova M, Morris L, Paul L (2013). Psychosocial interventions with cognitive-behavioral components for the treatment of cancer-related traumatic stress symptoms: a review of randomized controlled trials. J Cogn Psychother.

[30] Schell LK, Monsef I, Wöckel A (2019). Mindfulness‐based stress reduction for women diagnosed with breast cancer. Cochrane Database Syst Rev.

